# Successful treatment following early recognition of a case of Fournier’s scrotal gangrene after a perianal abscess debridement: a case report

**DOI:** 10.1186/s13256-018-1697-9

**Published:** 2018-06-27

**Authors:** Youwen Chen, Xueke Wang, Guoren Lin, Rukai Xiao

**Affiliations:** 1 Department of Urological Surgery, Chang Gung Memorial Hospital, 123 Avenue Xiafei, Xiamen, 361028 Fujian China; 2 Department of Urological Surgery, Chang Gung Memorial Hospital, No. 5 Fuxing Street, Guishan District, Taoyuan, Taiwan

**Keywords:** Fournier’s gangrene, Perianal abscess debridement, Necrotizing fasciitis, *Streptococcus agalactiae*

## Abstract

**Background:**

Fournier’s gangrene is an acute surgical emergency characterized by high mortality rates ranging from approximately 13% to 45%. Therefore, aggressive multidisciplinary management is necessary.

**Case presentation:**

A 29-year-old Asian man who had undergone surgical debridement at another hospital to treat a perianal abscess 5 days earlier was admitted to our hospital for severe scrotal and perianal pain, swelling, and high fever. A physical examination revealed a perianal abscess. Furthermore, the scrotum was gangrenous and exhibited extensive cellulitis in the perineum and bilateral inguinal area. Crepitations between the skin and fascia were palpable. A diagnosis of Fournier’s gangrene was made. The patient was treated with immediate surgical debridement under general anesthesia. He received broad-spectrum antibiotics, and debridement was repeated until the wound exhibited healthy granulation. Because both testes were severely exposed, they were transpositioned back into the scrotum 1 week after surgery. The patient was discharged on the 11th postoperative day.

**Conclusions:**

The mainstay of treatment for Fournier’s gangrene should include fluid resuscitation, broad-spectrum antibiotic therapy, intensive care, nutritional support, and early aggressive surgical debridement of all necrotic tissue.

**Electronic supplementary material:**

The online version of this article (https://doi.org/10.1186/s13256-018-1697-9) contains supplementary material, which is available to authorized users.

## Background

Fournier’s gangrene (FG) is a rare, synergistic, fulminant form of necrotizing fasciitis that involves the genital, perineal, and perianal regions [1]. Its mortality rate remains high. This condition continues to be a major challenge to the medical community despite enormous advances in both intensive medical treatment and antibiotic therapy. FG was first described in 1883 in a review of five patients as a fulminant spreading infection of the subcutaneous tissues and superficial fascia of the scrotum and penis [1]. The flora was described as polymicrobial, had a poorly defined portal of entry, and affected otherwise healthy young men. Since it was first described, understanding of this disease has improved considerably, and its epidemiology, clinical features, and pathogenesis have been well defined [1, 2].

Currently, FG is viewed as a potentially fatal condition that affects all ages and both sexes. It causes thrombosis in small vessels, obliterative endarteritis, and eventually skin and tissue necrosis [3]. Recent reports show that in the majority of cases, there are underlying etiological factors, including local trauma, surgery to the scrotum or perineum, periurethral or perianal sepsis, and retroperitoneal rupture of the intraabdominal viscera [1–3]. Predisposing factors believed to contribute to the development of this disease include diabetes mellitus, alcoholism, malignancies, immunosuppression, and liver and renal diseases [4]. The importance of perianal sepsis is further indicated by a recent review of the organisms involved in scrotal infections. In the majority of cases, aerobic and anaerobic bacteria are synergistically involved and originate in anorectal and urogenital trauma and/or infection [5].

FG is characterized by high mortality rates (range, ~ 13–45%) and represents an acute urological and surgical emergency [5]. In this severe complication, morbidity and mortality depend on early recognition and diagnosis, hemodynamic stabilization, effective antibiotic treatment, and urgent aggressive surgical debridement. In this report, we present a case of FG that occurred in a patient who had undergone perianal abscess debridement and was successfully treated following early recognition in our department despite the severity of the patient’s condition.

## Case presentation

A 29-year-old Asian man who had undergone surgical debridement at another hospital for a perianal abscess 5 days earlier was referred to the emergency room of Xiamen Chang Gung Hospital. The patient presented with continuous severe perianal and scrotal pain, scrotal swelling, and high fever (39.2 °C) of 3 days’ duration that had been aggravated for 1 hour. The patient was mildly obese, described himself as otherwise quite healthy, and had never been admitted to a hospital previously. He reported no significant chronic medical history, such as primary hypertension, any type of heart disease, disturbed microcirculation, peripheral neuropathy, diabetes mellitus, an impaired immune system, malignancies, leukemia, long-term administration of corticosteroids, liver cirrhosis, renal failure, urinary tract infection, or hemodialysis. The patient also reported no history of infectious diseases, such as tuberculosis, any type of hepatitis, or acquired immunodeficiency syndrome (AIDS). The patient’s medical history revealed no trauma, blood transfusion, other surgical procedures, or other serious event. He had not lived in an epidemic area and had no contact history of toxicity or radioactive exposure. The patient denied a family history of any inherited cancer. He did not smoke or consume alcohol and reported no other unhealthy lifestyle behaviors. The patient was a businessman by occupation and traveled for business most of the time.

A general physical examination on admission revealed that the patient was hypotensive (blood pressure, 92/63 mmHg) and tachycardic (heart rate, 117 beats/minute). No positive signs were found during the neurological, cardiopulmonary, and abdominal examinations. Neither pain around the kidney area with percussion nor tenderness along the bilateral ureteral approach was found. No bulging, tenderness, or mass was evident in the bladder area. A genital examination revealed a normal distribution of pubic hair and normal penile development without deformity, prepuce, penile ulceration, tenderness, induration, or neoplasms. No ectopia or secretions were found at the urethral orifice. A perianal abscess and hemorrhoids were identified upon full perineal examination (a previous surgical wound with obviously inflammatory secretions was detected 2.0 cm from the anal edge and approximately 1.5 × 2.5 cm in size), and the patient’s rectum was noted to be very tender. Furthermore, the scrotum was gangrenous with extensive cellulitis of the perineum and bilateral inguinal area and an increased skin temperature. No clear findings were obtained by palpation of the bilateral testis and epididymis.

Following a rectal examination, the patient rapidly became sweaty and unwell, and his scrotum became blue, swollen, and tense, with erythema extending into both groins. Crepitations between the skin and fascia were palpable. The patient noted a concomitant deterioration in his urinary stream and a degree of hesitancy. His white blood cell, red blood cell, and platelet counts were 23.1 × 10^9^/L, 4.11 × 10^12^/L, and 112 × 10^9^/L, respectively. Upon admission, his hemoglobin was 123 g/L, his ST-segment elevation was 84.0%, and his C-reactive protein level was 275.55 mg/L. The results of serology for both liver and renal function were normal. His coagulation and electrolyte profiles were normal. Computed tomography of the lower abdomen and pelvis (Additional files 1 and 2) revealed extensive emphysema around the testicles, epididymis, and perineal subcutaneous tissues. A diagnosis of FG complicating a perianal abscess was made.

**Fig. 1:**
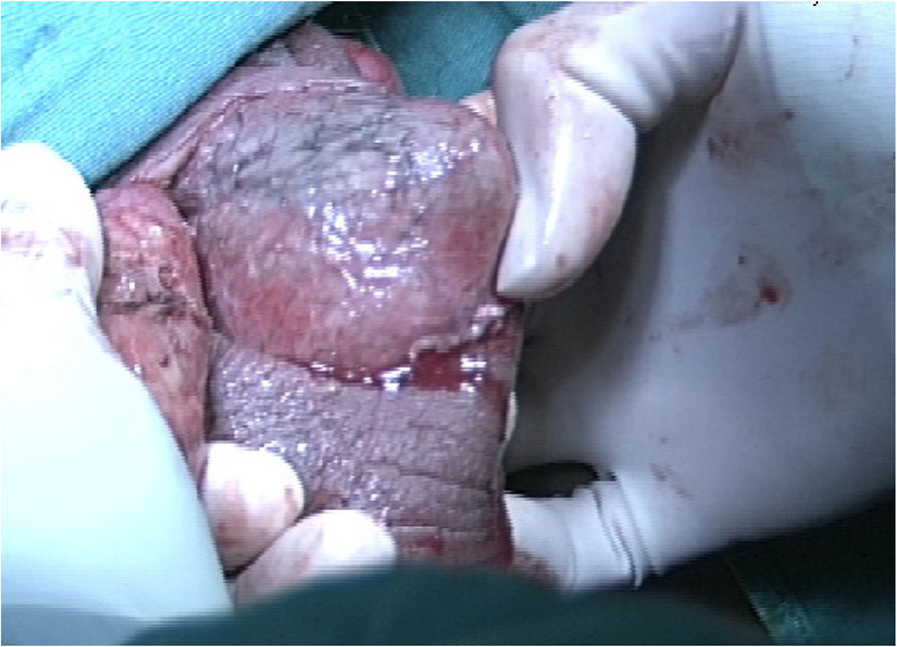
Preoperative image showing considerable edema and scrotal necrosis

**Fig. 2:**
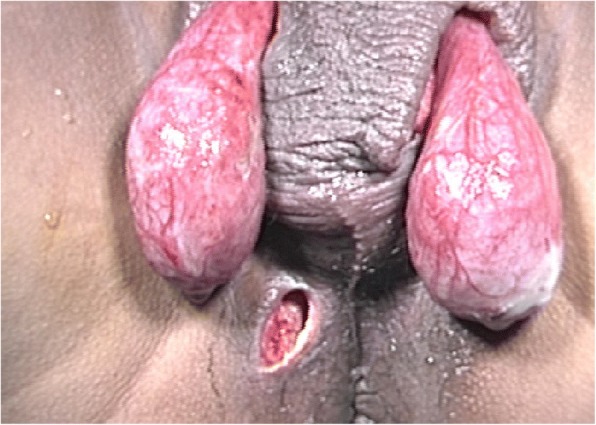
Intraoperative image of immediate surgical debridement in which open drainage from the scrotum and perianal region was achieved using three incisions. This procedure left both testes completely exposed

The patient underwent aggressive fluid administration, hemodynamic support, and intravenous antibiotic therapy. He was treated with immediate surgical debridement under general anesthesia (Fig. 1). Tissue cultures were obtained to isolate the responsible microorganisms. The necrotic skin in the scrotum and the perianal region was evacuated into an open drainage area using three incisions, leaving both testes exposed (Fig. 2). There was no damage to the testicles, spermatic cords, or external sphincter.

Blood and aerobic and anaerobic bacterial cultures were performed. Microorganisms were not found in the blood cultures. Tissue samples taken during the first two debridements revealed that the microbiological etiology of FG was polymicrobial. Aerobic *Streptococcus agalactiae*, *Staphylococcus haemolyticus*, and *Escherichia coli* as well as anaerobic peptostreptococci and *Prevotella corporis* were detected. Preoperative antibiotic treatment with combined broad-spectrum antibiotics (metronidazole 0.5 g every 8 hours and ceftriaxone 1 g every 12 hours for 6 days) was initiated and later adjusted to the culture sensitivity of the microbial isolates. The patient was therefore intravenously administered levofloxacin (0.3 g every 12 hours for 5 days).

The patient’s white blood cell count and C-reactive protein level gradually decreased. On the third postoperative day, his white blood cell, red blood cell, and platelet counts were 6.6 × 10^9^/L, 4.54 × 10^12^/L, and 190 × 10^9^/L, respectively. His hemoglobin was 135 g/L, his ST-segment elevation was 64.0%, and his C-reactive protein level was 100.24 mg/L. He had no fever thereafter.

**Fig. 3:**
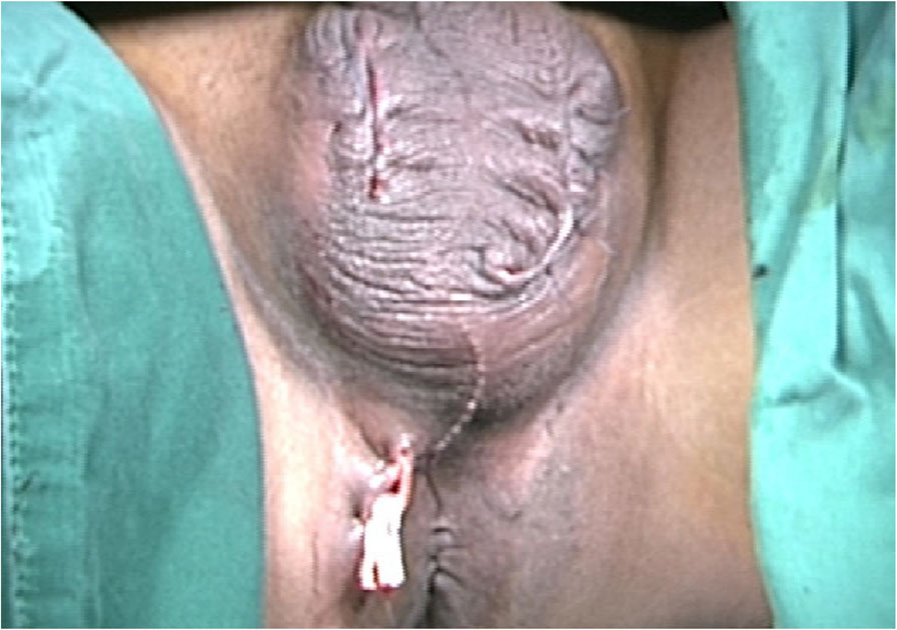
Image of both testicles following transpositioning into the scrotum on the eighth postoperative day. Split-skin closures were carried out using 3-0 nylon suture, and open drainage from the scrotum and perianal area was subsequently performed

Local wound care was administered using moist gauze dressing (i.e., normal saline) and was changed three times per day until healthy granulation tissue was observed. The patient underwent two subsequent surgical debridements. His infection gradually subsided, his gas gangrene resolved completely, and good granulation was present 1 week after surgery. Because both testicles were severely exposed, they were transpositioned into the scrotum on the eighth postoperative day. Split-skin closures (3-0 nylon suture) and open drainage from the scrotum and perianal area were subsequently performed (Fig. 3). The patient was discharged on the 11th postoperative day (with antibiotics adjusted to oral administration of levofloxacin 0.1 g twice daily).

The patient attended the outpatient department four times for follow-up. The bilateral scrotal wounds healed, and the stiches were removed on the 18th postoperative day (the first follow-up). The viability of both testicles was confirmed using Doppler ultrasound on the 21st postoperative day (the second follow-up) and at 3 and 10 months after discharge (the third and fourth follow-up examinations). The open drainage area from the scrotum and perianal area had healed at 10 months after discharge. The patient was healthy as usual, with no complaints or illness. His complete cell count and serum parameters of liver and kidney function were normal.

## Discussion

We present a case of FG that occurred in a patient who had undergone perianal abscess debridement and who was successfully treated following early recognition despite the severity of the patient’s condition. In this case, the possible etiology of the rectal abscess might be an unclean living habit, especially at times when the patient had hemorrhoids. A potentially inadequate perianal abscess debridement was considered the cause of FG in this case. An uncommon feature of this case is that *S. agalactiae* infection, contracted via sexual transmission, synergistically formed a subcutaneous abscess and resulted in FG.

FG is an unusual but very serious condition. It begins as a local infection that is caused by bacteria inhabiting the lower gastrointestinal tract or the perineum. FG is a polymicrobial infection of the perineoscrotal region that manifests as a rapidly progressing necrotizing fasciitis. The synergistic action of aerobic and anaerobic organisms plays a major role in the progressive course of the infection, which leads to an inflammatory response that spreads to the fascia, with resultant obliterative endarteritis, thrombosis of the cutaneous and subcutaneous vessels, and tissue necrosis [3–5]. This process can be facilitated by an impaired immune system, diabetes mellitus, alcoholism, malignancies, leukemia, long-term administration of corticosteroids, AIDS, liver cirrhosis, renal failure, and hemodialysis, all of which predispose a patient to developing FG [6, 7]. Diabetes mellitus is especially implicated and is present in approximately 65% of affected patients. It renders them vulnerable to infection in the setting of disturbed microcirculation, peripheral neuropathy, dysfunctional neutrophils, hypofunctional immunity, dehydration, and a systemic disturbance in carbohydrate metabolism [7–9].

FG affects both men and women of all ages, ranging from neonates to the very elderly. The mean age of patients ranges from approximately 40 to 50 years [8, 9]. FG after a perianal abscess debridement is rare. The morbidity and mortality in this severe complication depend on early recognition and diagnosis as well as aggressive surgical management.

FG is a rare disease, with approximately 400 cases reported in Europe and North America [6]. It was previously considered an idiopathic entity; however, recent reports indicate that in the majority of cases, there is an underlying etiological factor. Currently, its most common initial ports of entry are thought to be local trauma or the extension of a urinary tract or perianal infection [7–9]. With regard to the genitourinary tract, urethral strictures and transurethral instrumentation are the most frequent etiologies. Other causes include surgery to the penis, scrotum, or perineum; phlebitis of the dorsal vein of the penis; periurethral or perianal sepsis or retroperitoneal rupture of intraabdominal viscera; urethral calculi; bladder cancer infiltrating the urethra; and transrectal prostate biopsy [5–7]. Perforated acute appendicitis, diverticular perforation, carcinoma of the sigmoid colon and rectum, and dilation and internal hemorrhoids that were ligated with rubber bands have also been reported as etiologies of FG [8, 9]. The importance of anorectal sources of infection, including intersphincteric, perianal, and ischiorectal abscesses, especially when inadequately treated, was emphasized in a recent article [7]. In our patient, a potentially inadequate perianal abscess debridement was considered the cause of FG.

The most common clinical features of FG are perianal pain and swelling when the anorectal area is the port of entry and urinary retention and testicular or scrotal pain when the infection originates from the genitourinary tract [9–11]. Other systemic manifestations may include fever, tachycardia, electrolyte imbalances, and hyperglycemia. FG can rapidly develop into endotoxin shock, streptococcal toxic shock-like syndrome, disseminated intravascular coagulation, and multiple organ failure [10, 11]. Our patient was admitted to our hospital with severe perianal pain, scrotal edema, crepitus, high fever, tachycardia, electrolyte imbalances, and low blood pressure.

The bacteria most frequently isolated from FG are anaerobic *Bacteroides fragilis*, peptostreptococci, *Clostridium* and *Fusobacterium* spp., and aerobic *E. coli* and streptococci [9–11]. *S. agalactiae*, *S. haemolyticus*, *E. coli*, and peptostreptococci were identified in the pus in our patient. *S. agalactiae* belongs to group B streptococci and is one of the resident flora in the human vagina. It is present in approximately 20–30% of human vaginas and is rarely found in male genital regions; it is considered a sexually transmitted organism [11]. It is less virulent than members of group A streptococci but has received more attention because it can cause suppurative meningitis or sepsis in newborns and is an opportunistic pathogens in adults [11]. Because our patient was a sexually active man, we assumed that a sexually transmitted infection resulted in this organism entering his genital regions, where it synergistically formed a subcutaneous abscess.

There is general agreement in the literature regarding the initial treatment of patients with FG. All authors agree on the need for early vigorous treatment with local debridement of necrotic tissue and appropriate antibiotics. Once a diagnosis of FG has been established, rapid fluid resuscitation and the restoration of cardiopulmonary function have undisputed roles in the management of patients with sepsis for aggressive hemodynamic stabilization. Initial therapy includes parenteral broad-spectrum antibiotics to treat the infection. The broad-spectrum antibiotics should be changed or continued depending on culture findings. In the majority of cases, double- or triple-drug therapy with a combination of a third-generation cephalosporin, metronidazole, and an aminoglycoside for gram-negative aerobes and other organisms is recommended [7, 8]. The key to ensuring a successful outcome and survival of patients with FG is prompt and aggressive surgical intervention. The objective of this surgery is to remove the devitalized tissue to halt the progress of infection and to eliminate the systemic effects of necrotic material, toxins, and bacteria. Surgical debridement of necrotic tissue must be performed until the wound bed is clean and healthy.

Testes and spermatic cords are generally not affected by this disease, because they are supplied by the testicular artery [7, 8]. In our patient, both testicles were completely exposed to more efficiently and completely drain the scrotum. Then, the testicles were transpositioned into the scrotum on the eighth postoperative day because the infection had gradually subsided and healthy granulation was present. Randall [12] noted that rapid regeneration of the scrotum can be expected once the gangrenous tissue has been removed or has separated spontaneously. Given this natural tendency of the scrotum to regenerate, more elaborate procedures to provide cover for the testes appear to be unnecessary. However, such procedures may be helpful in shortening the period required to attain full healing; both spilt-skin grafts and subcutaneous pockets in the upper thigh have been used [12]. Nevertheless, in our patient, concerns regarding the future function of the testicles, testicular pain and atrophy, and temperature regulation were observed. Moreover, the viability of the testicles was confirmed using Doppler ultrasound several days postoperatively.

## Conclusions

Our patient was a young man with FG that developed following a perianal abscess debridement. The cause of the FG was considered to be a potentially inadequate perianal abscess debridement and a sexually transmitted *S. agalactiae* infection. Hospitalization for this disease is typically long, with a reported average of 6 weeks [10]. Mortality rates are highest in patients presenting with sepsis, diabetes mellitus, and late admission to the hospital [10, 11]. Because FG was recognized early in our patient and aggressive multidisciplinary treatment was administered, our patient survived and was discharged 11 days after admission. The keystones of an appropriate therapeutic approach include fluid resuscitation; broad-spectrum antibiotic coverage; intensive care; nutritional support; and, most important, repeated surgical debridement.

## Additional files

Additional file 1: Computed tomography of the lower abdomen and pelvis in the horizontal plane. (JPG 47 kb)

Additional file 2: Computed tomography of the lower abdomen and pelvis in the sagittal plane. (JPG 44 kb)

## Abbreviations

AIDS: Acquired immunodeficiency syndrome
FG: Fournier’s gangrene
