# Supplementary figures and images for: Successful treatment following early recognition of a case of Fournier’s scrotal gangrene after a perianal abscess debridement: a case report
Source: J Med Case Rep. 2018 Jun 27;12:193. doi: 10.1186/s13256-018-1697-9 (PMC6020361; doi:10.1186/s13256-018-1697-9)

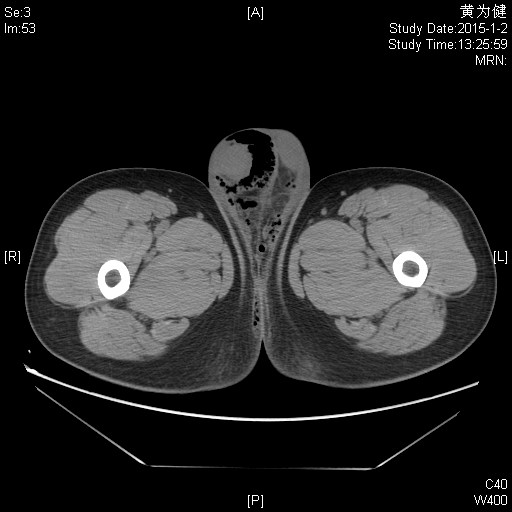

Supplement: Supplementary file 1 — Computed tomography of the lower abdomen and pelvis in the horizontal plane. (JPG 47 kb) [file 13256_2018_1697_MOESM1_ESM.jpg]

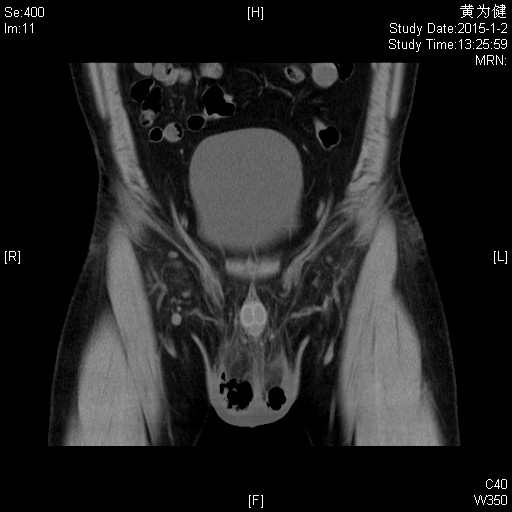

Supplement: Supplementary file 2 — Computed tomography of the lower abdomen and pelvis in the sagittal plane. (JPG 44 kb) [file 13256_2018_1697_MOESM2_ESM.jpg]
